# Cognitive Functions across the GNB3 *C825T* Polymorphism in an Elderly Italian Population

**DOI:** 10.1155/2013/597034

**Published:** 2013-10-22

**Authors:** Edoardo Casiglia, Nunzia Giordano, Valérie Tikhonoff, Giovanni Boschetti, Alberto Mazza, Sandro Caffi, Federica Guidotti, Patrizia Bisiacchi

**Affiliations:** ^1^Department of Medicine, University of Padova, 35128 Padova, Italy; ^2^MRC Unit for Lifelong Health and Ageing at UCL, London WC2B 4AN, UK; ^3^Department of Internal Medicine, Hospital of Rovigo, 45100 Rovigo, Italy; ^4^General Direction, University Hospital of Verona, 37126 Verona, Italy; ^5^Department of General Psychology, University of Padova, 35128 Pdova, Italy

## Abstract

To verify whether the *C825T* polymorphism of the GNB3 influences the response to neuropsychological tests, mini-mental state examination, digit span (DS), immediate and delayed prose memory, memory with interference at 10 and 30 seconds (MI 10 and 30), trail making tests (TMTs) A and B, abstraction task, verbal fluency (VF) test, figure drawing and copying, overlapping figures test and clock test were performed in 220 elderly men and women free from clinical dementia and from neurological and psychiatric diseases randomly taken from the Italian general population and analysed across the *C825T* polymorphism. The performance of DS, immediate and delayed prose memory, VF, and TMTs was worse in subjects who were TT for the polymorphism in comparison to the C-carriers. The performance of all tests declined with age. In the case of DS, immediate and delayed prose memory, MI 10 and VF, this trend was maintained in the C-carriers but not in TT. In the case of prose memory, of memory with interference, and of VF, schooling reduced the detrimental interaction between age and genotype. The *C825T* polymorphism of GNB3 gene therefore influences memory and verbal fluency, being additive to the effects of age and partially mitigated by schooling.

## 1. Introduction

The heteromeric guanine nucleotide-binding proteins (G-proteins) are composed of *α*, *β*, and *γ* subunits. The *β* subunit is a regulator of signal transduction receptors and effectors. The *C825T *single-nucleotide polymorphism in the guanine nucleotide-binding protein beta polypeptide 3 (GNB3) gene encoding for *β*3 subunit located on chromosome 12p13 has been found to be associated with essential hypertension [1] and obesity [2]. 

In recent literature, this polymorphism has also been associated with neurological and psychic conditions such as depression [3–6], dementia [7], dependence [8], dyskinesia [9], and neurologically determined functional diseases [10, 11] and with response to antidepressant [5] or antipsychotic [12] drugs. Nevertheless, all these studies have been performed on psychiatric patients or in subsets of selected subjects. To our knowledge, no one of the studies dealing with this association was population based.

Neuropsychological tests allow standard evaluation of cognitive functions. Memory, language, and executive functions can be assessed and quantified by numeric scores. General cognitive assessment can be performed by means of mini-mental state examination [13]. A comprehensive neuropsychological battery of validated *paper and pencil* tests is also relevant for exploring focally the areas of cognitive functions putatively related to cognitive decline [14–17] and is currently employed for assessing the cognitive ability [18].

It has also been found that poorly educated and elderly persons may show low scores at neuropsychological tests even without developing clinical evidence of dementia [19]. 

The aim of the present study was to verify whether, at a population level, the *C825T* polymorphism of the GNB3 influenced the response to neuropsychological tests. For this aim, a representative sample of a general population was used. Only subjects aged ≥60 years were included in the analysis, as it is known that the full expression of the genetic potential of GNB3 gene requires decades of life [2]. 

## 2. Methods

### 2.1. Study Cohort

The study cohort was represented by unselected elderly subjects free from clinical dementia and from neurological and psychiatric diseases, randomly taken from the Italian general population in the frame of the Last Evidences Of Genetic Risk factors in the Aged (LEOGRA) study, whose protocol has been diffusely described elsewhere [20–22]. In brief, all the adults of this geographic area were called; 1,663 of them (73%) adhered to the protocol, gave informed consent, and were recruited, screened and followed up for 10 years or until death at a special hospital unit. A cohort of 220 subjects aged 60–95 years, chosen according to a random number list and representative of the whole population, was taken into consideration for the here in described study on cognitive tests.

### 2.2. Anthropometrics

Weight was measured in kg with a mechanical device Astra (GIMA, Gessate, Italy), height in m. Body mass index was calculated in kg/m^2^ from the weight/squared height ratio. 

### 2.3. Questionnaires and Blood Exams

All subjects underwent a Rose's questionnaire [23] concerning personal data, lifestyle, smoking, quality of life, and personal and familial anamnesis. 

Blood for exams was taken in the morning after overnight fasting. Blood glucose was measured with the colorimetric method at 630 nm, plasma insulin with radioimmunoassay. Low-density-lipoprotein cholesterol was calculated with the Friedlander algorithm [24]. As a measure of insulin resistance, the homeostasis model assessment index was calculated from HOMA-R = (circulating insulin in *μ*U/mL) × (fasting blood glucose in mmol/l)/22.5.

### 2.4. Instrumental Exams

All subjects underwent blood pressure measurements in triplicate by trained medical doctors by means of an automatic Omron 705-IT device (Omron Europe, Hoofddorp, Netherlands); to minimize the alert reaction, the average of the last two measurements was taken into account for the analysis of data. Subjects also underwent a standard electrocardiogram blindly codified according to the Minnesota code, a 2D-echocardiogram recorded according to the American Society of Echocardiography and the Penn convention (Megas device, Esaote, Firenze, Italy) and a spirometry (Pony Spyrometer class I, type B, Cosmed, Rome, Italy). Murmurs at neck were detected with the auscultatory method.

### 2.5. GNB3 Polymorphism Assessment

Genomic DNA was isolated from whole blood collected in ethylenediamine tetraacetic acid tubes using a blood DNA Prep Plus spin-column system, according to the protocol provided by the manufacturer (A&A Biotechnology, Gdansk, Poland). For the determination of the *C825T* polymorphism of the GNB3 gene, DNA was extracted from cellular blood components by the salting-out method and amplified; 80 ng of genomic DNA were then subjected to 35 rounds of specific amplification using 5′ TGA CCC ACT TGC CAC CCG TGC 3′ (sense primer) and 5′ GCA GCA GCC AGG GCT GGC 3′ (antisense primer). After denaturation at 94°C, DNA was amplified using 0.5 U of Taq polymerase at 94°C for 1 minute, 61°C for 1 minute, and 72°C for 1 minute. After a final extension for 7 minutes, polymerase chain reaction products were digested with BseDI (Fermentas), separated on 2.5% agarose gels, and visualized under UV illumination. The undigested product (TT genotype) has a size of 268 bp; complete digestion (CC genotype) results in bands of 116 and 152 bp, respectively [25]. The three genotypes were scored after running on a 2.5% agarose gel with 10 mg/mL ethidium bromide.

### 2.6. Neuropsychological Assessment

The evaluation consisted of a battery of validated tests relevant for exploring areas of cognitive functions potentially related to cognitive decline. Fourteen tests were selected in order to assess participant abilities in cognitive processing and grouped according to the function mainly involved:

- general functioning: mini mental state examination [26] (MMSE) and abstraction;
- memory tests: digit span, immediate and delayed memory, and memory with interference;
- executive tests involving one or more executive functions: overlapping figures, verbal fluency, trail making tests A and B (TMTs A and B);
- visuospatial abilities: spontaneous drawing, figure copying, and clock test.

Abstraction was studied by detecting logical abilities looking for a concept overordinating two terms.

Digit span [27] consisted of memorization and repetition of a series of numbers. Long-term memory was assessed by tests of immediate and delayed prose memory [28] presenting a prose passage containing 30 words and assessing immediate verbatim recalls followed by a 10 min delayed verbatim recall. Working memory was assessed by tests of memory with interference at 10 and 30 seconds (MI10, MI30): the participants were requested to recall a consonant trigram after an interval delay during which they had to count backward starting from a 3-digit random number presented by the examiner immediately after the trigram. At the end of the interval delay of 10 and 30 seconds, the subject had to recall the trigram [29]. 

Verbal fluency [30] was detected requiring participants to generate names from a specified category in a fixed period of time in 3 trials 60 seconds each.

In the TMT A subjects were required to connect with line progressive numbers, in the TMT B progressive numbers and letters [31, 32]. 

The overlapping figure was composed of 50 objects integrated into one perceptual unit [33, 34].

In the clock test the participant was instructed to draw a clock indicating 2 : 45, setting the hands and numbers on the face “so that a child could read them”. The instructions could be repeated until they were clearly understood, but once the subject began to draw, no further assistance was allowed.

The entire battery of tests was administered in a single session which took approximately two hours to complete. The results of the neuropsychological battery were compared to the normative sample for Italian subjects aged 60 years or over described by Mondini et al. [14]. The MMSE score was compared to the Italian normative scores described by Magni et al. [26]. 

### 2.7. Ethical Considerations

The investigation conformed to the principles outlined in the Declaration of Helsinki and institutional guidelines and was approved by the local Ethics Committee. Before the study and after consulting his/her own general practitioner, each subject accepted and signed an informed consent.

### 2.8. Statistical Analysis

A preliminary power analysis based on previous experience of the same laboratory [20, 21] and on previous specific literature [3–11] showed that 98 cases overall and 20 cases in each group were sufficient to avoid *β*-error with a desired statistical power of 0.08 and a probability level of 0.05. For this purpose, the CT and TT genotypes were considered together in a dominant genetic model for post hoc comparison, as the T allele has been found to exert a dominant effect [35]. To compare C-carriers and TT subjects, a Cohen's d of 0.2 to 10.1 was considered, leading to *a priori* samples ≤36 cases per group.

Continuous variables were expressed as mean ± standard deviation and compared between groups with analysis of covariance; 95% confidence intervals were also shown for unadjusted values of the score of the neuropsychological tests across the *C825T* polymorphism of GNB3 gene. Categorical variables were expressed as percent rate and compared with the Pearson *χ* ^2^ test. Non-normally distributed items were previously transformed into logarithm. The same procedure was done in order to make items independent of each other in multiple regression. 

The correlation with a continuous dependent variable was performed by stepwise multiple regression analysis, using both continuous and categorical items as independent variables; generating odds ratios and 95% confidence intervals (CI). Regression with categorical variables was performed logistically, also generating relative risks (RR) and 95% CI.

## 3. Results

### 3.1. Descriptive Statistics

The general characteristics of the cohort are shown in Table 1. In Table 2, the mean values of the test scores are summarized and compared to the normative values for Italian subjects of the same age and education. 

According to the *C825T* polymorphism of the GNB3 gene, 45.7% of subjects were CC, 36.3% CT, and 18.0% TT. This distribution was in line with that of Italian general population [2, 21, 35] and respected the Hardy-Weinberg equilibrium. The multiple regression of neuropsychological tests with age and education across the *C825T* polymorphism of GNB3 gene is shown in Table 3. The *C825T* polymorphism of GNB3 gene entered the model of analysis of variance for digit span (*F* = 9.76, *P* < 0.002), immediate (*F* = 5.56, *P* < 0.02) and delayed prose memory (*F* = 5.77, *P* < 0.02), MI10 (*F* = 3.26, *P* < 0.05), verbal fluency (*F* = 10.46, *P* < 0.002), and TMT A (*F* = 6.04, *P* < 0.01) and B (*F* = 4.60, *P* < 0.02). The unadjusted scores of the tests across the polymorphism are summarized in Table 4.

Blood pressure or diagnosis of arterial hypertension, heart rate, body mass index or diagnosis of overweight, blood lipids or diagnosis of hypercholesterolaemia or hypertriglyceridaemia, blood glucose or diagnosis of diabetes, serum uric acid or diagnosis of hyperuricaemia, left ventricular mass index or diagnosis of left ventricular hypertrophy, history of cardiovascular disease, intake of ethanol or caffeine, and smoking did not influence either the response to the neuropsychological tests or their scores across the *C825T* polymorphism of GNB3 gene.

### 3.2. General Functioning

**Figure 1:**
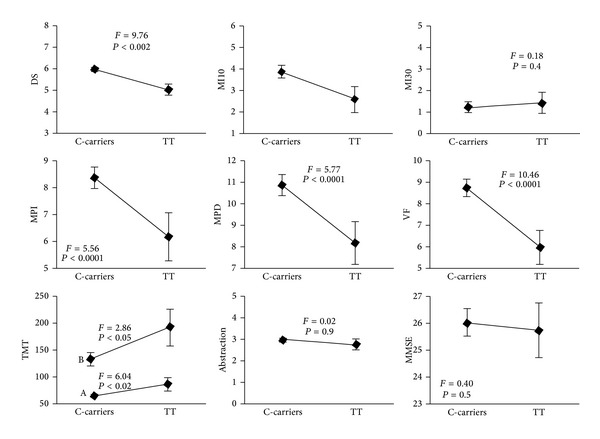
Scores of the neuropsychological tests in relation to *C825T* polymorphism of GNB3 gene in 220 subjects from general population. MI: memory with interference at 10 and 30 seconds, IPM and DPM: immediate and delayed prose memory, VF: verbal fluency, TMTs: trail making tests A and B, MMSE: mini-mental state examination.

MMSE scored in average 26.1 ± 3.9 (CI 25.5–26.7) and was inversely related with age (*r* = −0.23, *P* < 0.0001) and not different across the *C825T* polymorphism of the GNB3 gene either before or after adjustment for age and education (Figure 1). The RR for having a low performance at MMSE (score <24) was 1.16 (CI 1.08–1.23) in the whole cohort; after further stratification, a significant RR was found in TT subjects (1.15, CI 1.06–1.26) but not in those having CC or CT genotype.

The score of the abstraction task was related inversely with age; direct regression with years of school was present both in the whole cohort (Table 3) and separately in the C-carriers (*r* = 0.21, *P* < 0.001) and in the TT subjects (*r* = 0.28, *P* < 0.02). Even after adjustment for age and education, the score was not different across the *C825T* polymorphism (Figure 1). 

### 3.3. Memory

**Figure 2:**
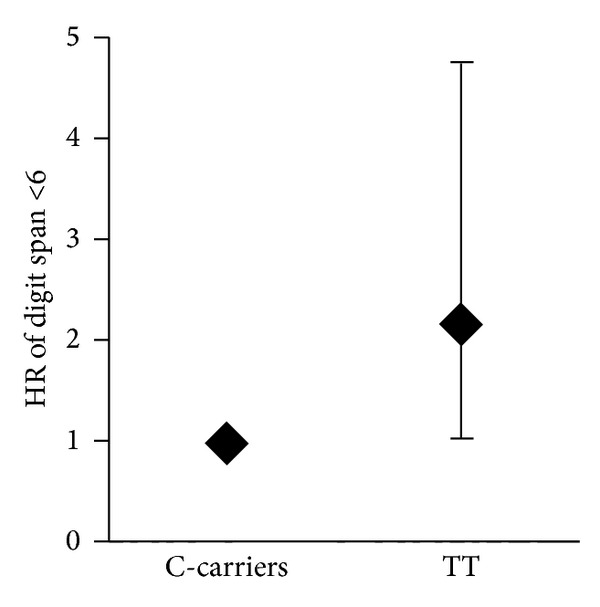
Relative risk (RR) of performing low digit span in relation to *C825T* polymorphism of GNB3 gene; 95% confidence intervals are also shown.

**Figure 3:**
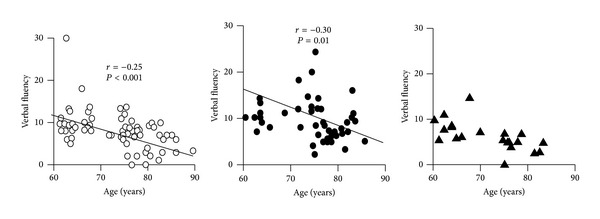
Regression of age with the memory tests in 220 subjects from general population.

In our cohort of elderly subjects, the digit span was inversely related with age, every year of age producing 7% decrease of the score (Table 3). The age-adjusted digit span across the *C825T* polymorphism of GNB3 gene is shown in Figure 1. After stratification, any relationship of digit span with age was no longer detectable in TT subjects (Figure 3). The age-adjusted relative risk of having a performance <6 at digit span was more than double for subjects carrying the TT polymorphism in comparison to the C-carriers (Figure 2). The digit span was not related with years of school in any of the polymorphisms. 

The immediate prose memory score was inversely related with age in the entire cohort (Table 3) and selectively in CC and CT but not in TT subjects (Figure 3). The scores adjusted for age and education across the *C825T* polymorphism are shown in Figure 1. Education did not modify either the score or its interaction with the T mutation.

The delayed prose memory score was related inversely with age and directly with years of schooling (Table 3). Regression with education was also present separately in the C-carriers (*r* = 0.35, *P* = 0.03) and in the TT subjects (*r* = 0.70, *P* = 0.02), while that with age was lost in TT (Figure 3). The scores adjusted for age and education across the *C825T* polymorphism of GNB3 gene are shown in Figure 1. 

The score of MI10 was inversely related with age (−17% for every year). Although the score was not different across the *C825T* polymorphism (Table 3 and Figure 1), any regression with age was lost in CT and TT subjects. Years of school had no effect (Table 3). The performance to this test was quite low, due to many subjects—particularly carrying the TT polymorphism—who scored 0 (Figure 3).

The score of MI30 was directly related with education in the whole cohort (Table 3) and in the C-carriers (*r* = 0.30, *P* < 0.0001) but not in the TT subjects and was not different across the GNB3/*C825T* polymorphism either before or after adjustment for age and education (Figure 1). Inverse regression with age was found (*r* = −0.07, *P* < 0.01), but disappeared after the introduction of the years of education. The performance to this test was quite low due to many subjects—particularly among the TT—who scored 0 (Figure 3).

### 3.4. Executive and Visuospatial Functions

The overlapping figure test was inversely related with age. After adjusting for age and education, its score was not different in C-carriers (17.4 ± 9.6) and in TT (17.7 ± 10.5, *P* = 0.37). In the C-carriers only, regression with years of education (*r* = 0.92, *P* < 0.001) not affecting the regression with age was observed. 

**Figure 4:**
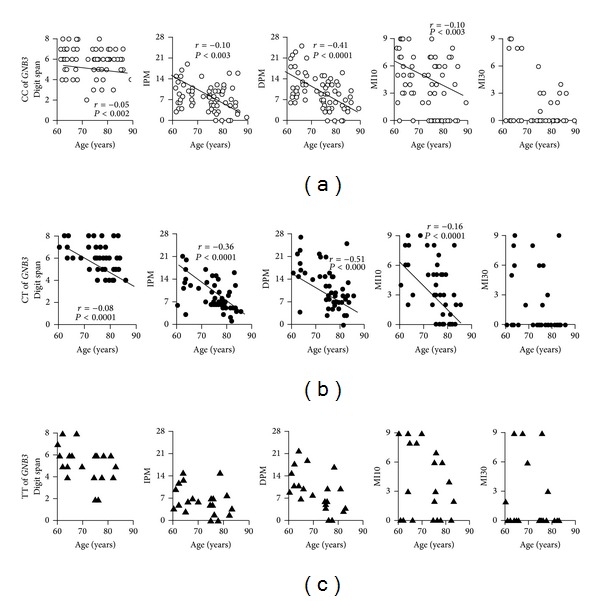
Regression of age with the neupsychological tests in 220 subjects from general population.

The score of verbal fluency was inversely related with age, with exclusion of the TT subjects, where no correlation was detectable (Figure 4). It was also directly related with years of schooling in the C-carriers only (*r* = 0.26, *P* < 0.05). After adjustment for age and education, it resulted to be 30% lower in TT subjects than in C-carriers (Figure 1). 

The TMTs were directly related with age and—limitedly to the C-carriers—inversely related with years of schooling (*r* = −2.28, *P* = 0.04; *r* = −6.26, *P* < 0.004, resp.). After adjustment for age and education, they were significantly higher in the TT subjects than in C-carriers (Figure 1). Adding education did not abolish the regression of the tests with age and with the *C825T* polymorphism of GNB3 gene.

The clock test score was inversely related to age, while no regression with genotype and education was found.

## 4. Discussion

In this cohort of general population, the *C825T* polymorphism of GNB3 gene resulted to be associated with cognitive functions. The TT pattern was inconvenient and leads to lower performance in many tests. Age was the main covariable of the interaction between genotype and performance. Schooling partially attenuated the effect of having unfavourable genotype. 

All tests of memory but MI30 were inversely related with age, but this effect was not homogenous, being unequally distributed across the *C825T* polymorphism of the GNB3 gene. 

Both short-term and long-term memory functions were *C825T* dependent, being carried out with lower performance by the TT subjects (digit −10.5%, IPM −19.5%, and DPM −9.7%), and in multiple logistic analysis the risk of having a limited performance at the digit span doubles in TT subjects. Not only this, but in TT subjects the performance of memory tests did not depend on age, probably indicating a genetically controlled acceleration of ageing. 

Education limited but did not abolish the effect of TT homozygosis on immediate and delayed prose memory, but not on digit span.

The TMT A and verbal fluency were influenced by the *C825T* polymorphism of the GNB3 gene, being worse in the TT genotype (an effect which was partially prevented by education), while the other executive functions (TMT B, figure drawing and copying, and overlapping figure test) were not different across the polymorphism. Verbal fluency worsened with increasing age in the C-carriers only, while the TT had a low score independently of age. Education improved the fluency in the C-carriers but not in the TT. This indicates an influence of the *C825T* polymorphism on the executive and visuospatial abilities.

To our knowledge, no previous data are available in the literature about the interactions between cognitive tests and the *C825T* polymorphism of GNB3 gene at a population level. Our results show that the TT homozygosis is unfavorable from a cognitive point of view. This suggests that memory and executive functions may in part be inherited, while abstractive and visuospatial abilities are not.

The reason of lower cognitive ability in subjects with the TT homozygosis can only be object of speculation, due to paucity of data in literature. Subjects with the *825T* mutation have lower striatal dopamine transporter availability, which is in turn regulated by presynaptic G-protein coupled autoreceptors [36]. The striatal dopamine system has recently been considered to involve circuits that participate in the integration of cognitive activities and reward responses through the corticothalamic-basal gangliancortical loop [37]. The striatum is reduced in conditions associated with cognitive impairment [38], and a reduced striatal activity is actually associated with cognitive fatigue [39] and with cognitive symptoms accompanying neurodegenerative conditions [40].

In our cohort considered as a whole, the cognitive pattern was mildly worse than expected from Italian normative data [35] probably because the TT subjects were more represented in our cohort (15%) than in the European population (8.1–8.4%) [41, 42]. The unfavorable cardiovascular pattern which is typical of the LEOGRA population [20, 21], and which in part derives in turn from the T mutation [2], could also account for impaired cognition.

A limitation of the study is represented by the fact that sample sizes were suboptimal, although they are representative of general population and ratified by power analysis.

## Figures and Tables

**Table 1:** General characteristics of the study cohort. Mean ± standard deviations are provided for continuous variables.

Items		*C825T* polymorphism of GNB3 gene		
	All subjects (*n* = 220)	CC subjects (*n* = 109)	CT subjects (*n* = 69)	TT subjects (*n* = 42)
Age (years)	73.2 ± 7.7	64.2 ± 9.2	64.9 ± 11.1	60.1 ± 8.7*
Males (%)	44.4	44.1	37.9	43.8
BMI (kg/m^2^)	26.6 ± 3.4	26.9 ± 3.3	27.0 ± 3.7	27.1 ± 4.4
LVMI (kg/m^2^)	119.7 ± 29.9	124.5 ± 36.2	126.7 ± 33.6	117.4 ± 36.0
LVH (%)	59.9	55.2	62.5	50.0
SBP (mmHg)	159.8 ± 21.7	159.9 ± 24.0	162.1 ± 22.1	152.8 ± 24.5
DBP (mmHg)	90.0 ± 10.6	89.7 ± 10.6	89.5 ± 9.7	88.6 ± 10.2
Heart rate (bpm)	68.1 ± 9.6	69.2 ± 9.8	67.0 ± 8.5	68.6 ± 10.7
Coronary artery disease (%)	31.6	37.1	28.6	22.2
Haematocrit (%)	41.7 ± 3.4	41.8 ± 3.4	41.0 ± 3.4	41.4 ± 3.7
Sedimentation rate (mm/h)	11.8 ± 9.7	11.7 ± 10.3	11.0 ± 8.0	12.3 ± 8.8
Blood glucose (mg/dL)	104.2 ± 18.5	103.4 ± 15.5	100.8 ± 16.4	107.9 ± 35.3
Serum uric acid (mg/dL)	4.9 ± 1.3	5.0 ± 1.2	4.7 ± 1.2	4.9 ± 1.4
LDL-C (mg/dL)	159.1 ± 37.1	158.7 ± 37.1	157.9 ± 33.7	158.2 ± 36.5
HDL-C (mg/dL)	46.9 ± 10.7	45.9 ± 11.1	47.3 ± 10.7	45.2 ± 11.5
TG (mg/dL)	119.7 ± 64.4	115.3 ± 61.9	114.7 ± 59.0	128.5 ± 61.2
Circulating cortisol (*μ*g/dL)	18.2 ± 4.9	18.3 ± 4.8	17.7 ± 4.5	18.7 ± 5.1
Circulating T3 (ng/dL)	116.3 ± 22.5	113.5 ± 21.2	110.4 ± 20.8	118.0 ± 26.5
Circulating T4 (*μ*g/dL)	8.5 ± 1.6	8.7 ± 2.0	8.4 ± 1.6	8.6 ± 1.8
Plasma TSH (mlU/L)	1.8 ± 1.5	1.9 ± 1.9	1.8 ± 1.4	1.5 ± 0.8
Circulating insulin (*μ*U/mL)	7.7 ± 5.7	7.6 ± 6.1	7.5 ± 4.9	8.6 ± 5.6
HOMA-R	2.0 ± 1.7	20 ± 1.8	2.0 ± 1.6	2.3 ± 1.8
Apolipoprotein B/A ratio	0.8 ± 0.2	0.7 ± 0.2	0.7 ± 0.2	0.8 ± 0.2
Education (years of school)	6.8 ± 2.9	6.1 ± 2.5	6.4 ± 2.8	6.3 ± 3.0
Hip-to-waist ratio	0.9 ± 0.1	0.9 ± 0.1	0.9 ± 0.1	0.9 ± 0.1

HOMA-R: homeostasis model assessment index; BMI: body mass index; LVMI: left ventricular mass index; SBP and DBP: systolic and diastolic blood pressure; LDL-C and HDL-C: low-density and high-density-lipoprotein cholesterol, TSH: thyrotropin-stimulating hormone; HOMA: homeostasis model assessment. *P < 0.02 TT versus the other two polymorphisms.

**Table 2:** Mean scores of the neuropsychological tests recorded in the study cohort. Italian normative scores for the same age and education are also shown [28].

Tests	Values from the cohort (*n* = 220)	Normative scores (*n* = 63)	*P* value
Digit span score	5.9 ± 1.4	5.5 ± 1.0	0.06 (ns)
Immediate prose memory score	9.0 ± 4.5	11.1 ± 3.4	<0.001
Delayed prose memory score	10.5 ± 5.8	13.8 ± 4.3	<0.0001
MI 10 score	3.7 ± 3.1	5.6 ± 2.5	<0.0001
MI 30 score	1.4 ± 2.5	5.3 ± 2.2	<0.0001
Trail making test A (seconds)	70.8 ± 35.0	77.9 ± 38.2	0.7 (ns)
Trail making test B (seconds)	159.4 ± 64.7	200.6 ± 84.0	<0.001
Verbal fluency score	8.4 ± 4.0	11.1 ± 2.9	<0.0001
Abstraction score	2.8 ± 2.1	4.5 ± 1.7	<0.0001
Overlapping figure score	17.9 ± 9.1	30.4 ± 7.0	<0.0001
Figure copying score	1.6 ± 0.6	1.5 ± 0.5	0.3 (ns)
Figure drawing score	1.5 ± 0.7	1.9 ± 0.3	<0.0001
Clock drawing task score	6.6 ± 3.6	8.1 ± 2.3	<0.003

MI: memory with interference; ns: nonsignificant difference.

**Table 3:** Multiple regression of the neuropsychological tests with age, education, and *C825T *polymorphism of GNB3 gene.

Tests	Age (years)		Education (years)		*C825T* of GNB3 (TT versus C-carriers)	
	*r* (SE)	*P*	*r* (SE)	*P*	*r* (SE)	*P*
MMSE	−0.21 (0.04)	<0.0001	0.07 (0.09)	0.4 (ns)	−0.15 (9.24)	0.5 (ns)
Digit span	−0.05 (0.01)	<0.001	0.04 (0.04)	0.3 (ns)	−0.30 (0.10)	<0.005
IPM	−0.17 (0.05)	<0.0001	0.44 (0.12)	<0.0001	−0.73 (0.32)	0.02
DPM	−0.36 (0.06)	<0.0001	0.58 (0.14)	<0.0001	−0.77 (0.32)	<0.05
MI 10	−0.14 (0.03)	<0.001	0.19 (0.08)	0.02	−0.18 (0.23)	0.4 (ns)
MI 30	−0.03 (0.04)	0.3 (ns)	0.35 (0.07)	<0.0001	0.10 (0.19)	0.3 (ns)
Verbal fluency	−0.19 (0.04)	<0.0001	0.23 (0.11)	<0.05	−0.80 (0.30)	<0.005
TMT A	1.32 (0.44)	<0.005	−2.47 (1.04)	<0.03	7.96 (3.12)	<0.01
TMT B	2.10 (1.02)	<0.05	−6.63 (2.47)	<0.01	18.80 (9.89)	0.06 (ns)
Figure drawing	−0.02 (0.01)	<0.01	0.01 (0.02)	0.9 (ns)	−0.07 (0.05)	0.1 (ns)
Figure copying	−0.03 (0.01)	<0.0001	0.04 (0.02)	0.4 (ns)	−0.07 (0.04)	0.1 (ns)
Abstraction	−0.09 (0.02)	<0.0001	0.22 (0.05)	<0.0001	0.01 (0.14)	0.9 (ns)
Overlapping figure	−0.58 (0.09)	<0.0001	1.00 (0.22)	<0.0001	−0.59 (0.61)	0.3 (ns)
Clock test	−0.15 (0.04)	<0.0001	0.16 (0.09)	0.1 (ns)	-0.46 (0.26)	0.1 (ns)

*r*: coefficient of regression; SE: standard error of the coefficient. MMSE: mini-mental state examination; IPM and DPM: immediate and delayed prose memory; MI 10 and MI 30: memory with interference at 10 and 30 seconds; TMTs: trail making tests; ns: nonsignificant.

**Table 4:** Unadjusted values of the score of the neuropsychological tests across the *C825T* polymorphism of GNB3 gene. The C-carriers were also cumulated.

Tests	CC (*n* = 109)	CT (*n* = 69)	C-carriers cumulated (*n* = 178)	TT (*n* = 42)
MMSE score	25.7 ± 4.1 (24.8–26.6)	26.5 ± 2.0 (25.9–27.3)	25.9 ± 3.7 (25.3–26.6)	26.6 ± 3.1 (25.4–27.9)
Digit span score	5.9 ± 1.4 (5.5–6.9)	6.1 ± 1.2 (5.8–6.4)	5.9 ± 1.4 (5.6–6.1)	5.3 ± 1.5 (4.6–5.9)
Immediate prose memory score	7.8 ± 4.6 (6.8–8.9)	8.6 ± 4.4 (7.4–9.9)	7.9 ± 4.4 (7.2–8.8)	7.2 ± 4.5 (5.3–9.0)
Delayed prose memory score	10.1 ± 5.9 (8.8–11.4)	11.2 ± 5.9 (9.5–12.8)	9.2 ± 5.8 (9.2–11.3)	9.9 ± 5.8 (7.5–12.3)
MI 10 score	3.8 ± 3.0 (3.1–4.5)	3.6 ± 3.0 (2.7–4.4)	3.1 ± 3.0 (3.1–4.2)	3.3 ± 3.5 (1.8–4.7)
MI 30 score	1.0 ± 2.4 (0.5–1.5)	1.2 ± 2.6 (0.5–2.0)	1.0 ± 2.3 (0.6–1.4)	1.6 ± 3.1 (0.3–2.9)
Verbal fluency score	8.0 ± 4.1 (7.1–9.0)	9.4 ± 4.2 (8.1–10.6)	8.5 ± 4.3 (7.7–9.3)	6.8 ± 3.1 (5.6–8.1)
TMT A (seconds)	69.1 ± 30.8 (61.3–76.9)	68.7 ± 34.0 (58.9–78.6)	69.3 ± 33.1 (62.7–75.8)	79.4 ± 51.1 (55.5–103.4)
TMT B (seconds)	168.6 ± 77.0 (140.9–196.4)	147.3 ± 55.3 (124.9–169.6)	158.5 ± 67.1 (140.2–176.8)	166.3 ± 51.0 (119.1–213.5)
Figure copying score	1.6 ± 0.6 (1.4–1.7)	1.8 ± 0.5 (1.6–1.9)	1.6 ± 0.6 (1.5–1.8)	1.6 ± 0.6 (1.3–1.9)
Figure drawing score	1.4 ± 0.7 (1.2–1.5)	1.7 ± 0.5 (1.6–1.9)	1.5 ± 0.6 (1.4–1.6)	1.4 ± 0.7 (1.1–1.7)
Abstraction score	2.7 ± 2.1 (2.2–3.2)	2.7 ± 2.0 (2.2–3.3)	2.8 ± 2.0 (2.5–3.1)	3.4 ± 2.3 (2.5–4.4)
Overlapping figure score	17.3 ± 9.9 (15.1–19.5)	18.3 ± 8.5 (15.9–20.7)	17.3 ± 9.5 (15.6–19.1)	18.7 ± 10.1 (14.5–22.8)
Clock test score	6.8 ± 3.6 (6.0–7.6)	6.5 ± 3.7 (5.5–7.5)	6.0 ± 3.6 (6.0–7.3)	6.3 ± 3.5 (4.9–7.8)

MMSE: mini-mental state examination; MI 10 and MI 30: memory with interference at 10 and 30 seconds; TMTs: trail making tests.

## Abbreviation

MMSE: Mini Mental state examination
DS: Digit Span
IPM and DPM: Immediate and delayed prose memory
MI 10 and MI 30: Memory with interference at 10 and 30 seconds
TMTs: Trail making tests.
